# Clinical Outcomes Following Open Olecranon Bursa Excision for Septic and Aseptic Olecranon Bursitis: An Observational Study

**DOI:** 10.7759/cureus.43696

**Published:** 2023-08-18

**Authors:** Nicholas B Pohl, Parker L Brush, Gregory R Toci, Jeremy T Heinle, Anna Thomas, Joshua Hornstein, Daren Aita, Pedro Beredjiklian, Brian Katt, Daniel Fletcher

**Affiliations:** 1 Division of Hand Surgery, Rothman Orthopaedic Institute, Philadelphia, USA; 2 Orthopaedic Surgery, Rothman Orthopaedic Institute, Philadelphia, USA; 3 Orthopaedic Surgery, Philadelphia College of Osteopathic Medicine, Philadelphia, USA; 4 Division of Sports Medicine, Rothman Orthopaedic Institute, Philadelphia, USA; 5 Division of Hand Surgery, Hackensack Meridian Ocean Medical Center, Brick Township, USA

**Keywords:** complications, outcomes, bursectomy, aseptic, septic, olecranon bursitis

## Abstract

Background and objective

Olecranon bursitis (aseptic or septic) is caused by inflammation in the bursal tissue. While it is typically managed with conservative measures, refractory cases may indicate surgical intervention. There is currently limited research about outcomes following bursal excision for both septic and aseptic etiologies. In light of this, the purpose of this study was to determine if patients experienced improvement following surgical olecranon bursa excision and to compare outcomes between septic and aseptic forms.

Materials and methods

A retrospective review was performed involving patients who underwent olecranon bursa excision from 2014 to 2021. Demographic data, patient characteristics, surgical data, and outcome-related data were collected from the medical records. Patients were classified into subgroups based on the type of olecranon bursitis (septic or aseptic). Preoperative and one-year postoperative 12-item short-form survey (SF-12) results and range of motion (ROM) outcomes were evaluated for the entire cohort as well as the subgroups.

Results

We included 61 patients in our study and found significant improvement in the Physical Component Scale 12 (PCS-12) score for all patients (42.0 vs. 45.5, p=0.010) following surgery. However, based on subgroup analysis, the aseptic group improved in PCS-12 following surgery (41.5 vs. 46.8, p<0.001), but the septic group did not (43.6 vs. 40.5, p=0.277). No improvements were found in the Mental Component Scale 12 (MCS-12) scores following surgery in either group. Eighteen of the 61 patients experienced postoperative complications (29.5%), but only 6.5% required a second surgical procedure. Specifically, 14 of the 18 complications occurred in the aseptic group while two septic and two aseptic patients required additional surgeries. Elbow ROM did not change significantly after surgery but more patients were found to have full ROM postoperatively (83.0% to 91.8%, p=0.228).

Conclusion

Our findings suggest that patients with refractory olecranon bursitis, particularly if aseptic, tend to gain significant physical health benefits from open bursectomy.

## Introduction

Olecranon bursitis develops when the bursal cavity superficial to the olecranon becomes inflamed. While the exact incidence of olecranon bursitis is unknown since most cases are treated in an outpatient setting, it is estimated to represent 0.01%-0.1% of hospital admissions [1]. Olecranon bursitis is often categorized into aseptic or septic variants based on history and physical exam, but the culture of bursal fluid remains the gold standard to differentiate between the two [2,3]. The most common cause of olecranon bursitis is trauma, in both the septic and aseptic forms [3].

Treatment options for olecranon bursitis range from non-standardized conservative management, including compression, rest, ice, physical therapy, and non-steroidal anti-inflammatory drugs (NSAIDs), to more invasive management, such as needle aspiration, corticosteroid injection, incision and drainage (I&D), and surgical bursa excision [4]. Needle aspiration is used for septic olecranon bursitis for diagnosis and management with additional invasive treatment options if indicated [5]. Patients may opt for surgical treatment if conservative treatment fails, and it is important to determine if surgical treatment results in measurable improvements in patients’ health and function. Patient-reported outcome measures are often employed to quantify improvement following treatment, and their utilization has become increasingly prevalent in upper extremity research [6].

There is currently limited research assessing patient-reported outcomes following surgical treatment for olecranon bursitis [7]. Hence, the purpose of this study was to determine if patients experienced improvement following surgical olecranon bursa excision for both septic and aseptic olecranon bursitis and to compare outcomes of bursa excision between septic and aseptic olecranon bursitis. We hypothesized that patients would improve as a result of bursal excision and that there would be no difference in outcomes between the septic and aseptic forms.

## Materials and methods

After obtaining institutional review board approval from our institution, patients who underwent olecranon bursa excision from 2014 to 2021 were identified by a query related to the Current Procedural Terminology (CPT®) code 24105. Adult patients (≥18 years) who underwent open olecranon bursa excision with preoperative and one-year postoperative SF-12 scores were included in this study [7]. Patients with olecranon bursitis associated with symptomatic hardware and patients with incomplete medical records were excluded from our analysis.

Patient characteristics, demographics, surgical data, and outcomes were collected from the electronic medical records, which included age, sex, race, BMI, length of follow-up, smoking status, hand dominance, type of olecranon bursitis (septic or aseptic), prior treatments (corticosteroid injections, needle aspirations, and I&Ds), antibiotic use, concurrent triceps tendon repair, postop drain utilization, time to wound healing, readmission rate, postoperative complications, and need for additional surgery (major complication). Comorbidity data regarding the history of gout, rheumatoid arthritis, and diabetes mellitus were also collected. The distinction between septic or aseptic olecranon bursitis was based on the presence of preoperative bursal fluid culture, intraoperative tissue/fluid culture, or, in the absence of culture data, clinical findings of a previous multicenter study on septic bursitis [8]. These findings include erythema, tenderness, fevers, rigors, swelling, and wound drainage. Clinical diagnosis of septic bursitis was made by a fellowship-trained hand & wrist, shoulder & elbow, or sports medicine surgeon at the time of patient consultation. Among patients who received preoperative antibiotics, those without other positive septic criteria listed previously were deemed to be aseptic. Patients' active range of motion (ROM) data were categorized as having full ROM or not and whether or not an extension lag was present. The patients were deemed to have full ROM if they had a flexion-extension arc of at least 135 degrees and no extension lag or if they were qualitatively documented as having full ROM.

A delta value was collected for both Mental Component Scale 12 (MCS-12) and Physical Component Scale 12 (PCS-12) scores, which was defined as the one-year postoperative value minus the preoperative value. This delta PCS-12 value was compared to the minimal clinically important difference (MCID) of 5.4 as previously defined in the literature [9]. MCS-12 and PCS-12 scores were used in this study as they were the most commonly collected patient-reported outcomes for patients undergoing open bursectomy. Descriptive statistics included mean and standard deviation (SD) for continuous variables and percentages for categorical variables. Paired t-tests were used to compare preoperative and postoperative MCS-12 and PCS-12. Patients were then classified into subgroups based on the type of olecranon bursitis, - septic or aseptic - and the presence or absence of concomitant triceps repair. Two-sample t-tests were used to compare continuous data between groups and chi-square tests were utilized to compare categorical data between groups. A p-value less than 0.05 was considered statistically significant. All statistical analyses were performed using RStudio Version 4.0.2.

## Results

The database query identified 733 patients as having received an olecranon bursectomy during the study period. Eighty-five of those patients had complete preoperative and postoperative SF-12 data. Among them, we removed 21 patients with symptomatic hardware and three patients due to incomplete records, and the remaining 61 patients were included in the final analysis. The average age of the cohort was 57.6 ±14.0 years., and the majority were male (50, 82%), white (48, 78.7%), right-handed (51, 83.6%), and non-smokers (36, 59.0%). The comorbidity profile included 12 patients with gout (19.7%) and seven patients with rheumatoid arthritis (11.4%). There were no patients with a history of diabetes mellitus, and therefore this was removed from the analysis for correlation with septic or aseptic bursitis. Regarding previous treatments, eight patients had a prior corticosteroid injection (13.1%), one patient had a prior platelet-rich plasma injection (1.6%), 21 patients underwent a prior aspiration (34.4%), and two patients underwent prior in-office I&D procedures (3.3%). During olecranon bursa excision, 20 patients (32.8%) required concurrent triceps tendon repair. Of these, eight were complete triceps tendon tears, and all were within the aseptic group. The patients were followed up postoperatively with in-office examinations for an average of 87.8 days. Table 1 summarizes the patient demographics and characteristics.

**Table 1:** Patient demographics and characteristics * and bold text indicate statistical significance (p<0.05) BMI: body mass index; I&D: incision and drainage; SD: standard deviation

Variables	Overall (n=61)	Infectious etiology		P-value	Concomitant triceps tear		P-value
		Aseptic (n=48)	Septic (n=13)		Without repair (n=41)	With repair (n=20)	
Age, years, mean (SD)	57.6 (14.0)	56.1 (13.9)	63.4 (13.1)	0.062	59.4 (14.8)	54.0 (11.6)	0.030*
Sex - female, n (%)	11 (18.0%)	8 (16.7%)	3 (23.1%)	0.687	10 (24.4%)	1 (5.0%)	0.084
Race, n (%)				0.264			0.264
Black/African American	6 (9.8%)	5 (10.4%)	1 (7.7%)		1 (10.4%)	5 (7.7%)	
Other/unreported	7 (11.4%)	7 (14.6%)	0 (0.00%)		4 (14.6%)	3 (0.00%)	
White	48 (78.7%)	36 (75.0%)	12 (92.3%)		36 (75.0%)	12 (92.3%)	
BMI, n (%)	29.8 (5.44)	30.1 (5.09)	28.9 (6.73)	0.208	29.7 (5.40)	30.0 (5.66)	0.854
Smoking status, n (%)				0.563			1.000
Current smoker	11 (18.0%)	10 (20.8%)	1 (7.69%)		7 (17.1%)	4 (20.0%)	
Former smoker	14 (23.0%)	10 (20.8%)	4 (30.8%)		10 (24.4%)	4 (20.0%)	
Nonsmoker	36 (59.0%)	28 (58.3%)	8 (61.5%)		24 (58.5%)	12 (60.0%)	
Hand dominance, n (%)				0.521			1.000
Ambidextrous	1 (1.65%)	1 (2.08%)	0 (0.00%)		1 (2.44%)	0 (0.00%)	
Left	9 (14.8%)	6 (12.5%)	3 (23.1%)		6 (14.6%)	3 (15.0%)	
Right	51 (83.6%)	41 (85.4%)	10 (76.9%)		34 (82.9%)	17 (85.0%)	
Laterality - right, n (%)	38 (62.3%)	32 (66.7%)	7 (53.7%)	0.208	28 (68.3%)	10 (50.0%)	0.270
Previous injection, n (%)	10 (16.4%)	9 (18.8%)	1 (7.69%)	0.674	7 (17.1%)	3 (15.0%)	1.000
Previous aspiration, n (%)	23 (37.7%)	16 (33.3%)	7 (53.8%)	0.208	21 (51.2%)	2 (10.0%)	0.005*
Previous I&D, n (%)	2 (3.3%)	0 (0%)	2 (15.4%)	0.0426*	6 (14.6%)	2 (10.0%)	1.000
Concurrent triceps tendon repair, n (%)	20 (32.8%)	17 (35.4%)	3 (23.1%)				
Complete triceps tear	7 (35%)	7 (41.2%)	0 (0%)				
Partial triceps tear	13 (65%)	10 (58.8%)	3 (100%)				
Follow-up, days, mean (SD)	87.8 (120)	97.3 (133)	52.4 (30.5)	0.374	80.6 (141)	102 (58.3)	<0.001*

Of the 61 patients, 13 (21.3%) were diagnosed with septic olecranon bursitis, and 48 (78.7%) were diagnosed with aseptic olecranon bursitis. Patients with septic olecranon bursitis were more likely to have had a prior I&D (46.2% vs. 4.17%, p=0.001), but there were no other significant differences in patient demographics or disease characteristics between the groups (Table 1). Of the 13 patients with septic olecranon bursitis, seven (53.8%) were noted preoperatively to have swelling, seven (53.8%) had erythema, six (46.1%) had documented drainage, two (15.4%) had tenderness to palpation of the posterior elbow, two (15.4%) displayed rigors, and one (7.7%) was noted to have a fever. Five patients with septic olecranon bursitis had positive preoperative wound cultures, of which four grew *Staphylococcus aureus* and one grew *Staphylococcus lugdunensis.* Among the eight patients without preoperative culture data, one patient grew *Pseudomonas spp.* from intraoperative tissue collection, two patients grew *Staphylococcus aureus* from intraoperative tissue collection, two patients had no growth from intraoperative tissue collection after preoperative antibiotics, one patient presented with a chronically draining sinus and reported negative cultures from an outside institution, and two patients presented with chronically draining sinuses from an infected olecranon bursa. Table 2 details patient-reported and postoperative outcomes, and Table 3 shows the multivariate regression with delta PCS-12 as the dependent variable and aseptic versus septic bursitis as the independent variables.

**Table 2:** Patient-reported and postoperative outcomes * and bold text indicate statistical significance (p<0.05). ^1^Delta: postoperative minus preoperative. ^2^Paired t-test between the postoperative and preoperative values MCID: minimal clinically important difference; MCS: Mental Component Scale; PCS: Physical Component Scale; SD: standard deviation

		Overall (n=61)	Infectious etiology		P-value	Concomitant Triceps Tear		P-value
			Aseptic (n=48)	Septic (n=13)		Without repair (n=41)	With repair (n=20)	
MCS-12 score	Preoperative, mean (SD)	56.0 (7.6)	57.0 (7.1)	52.6 (8.4)	0.062	55.2 (7.8)	57.8 (6.8)	0.155
	Postoperative, mean (SD)	54.3 (7.9)	55.4 (6.9)	50.1 (10.3)	0.091	54.5 (7.8)	53.9 (8.5)	0.951
	Delta^1^,mean (SD)	-1.8 (9.9)	-1.6 (9.4)	-2.6 (11.9)	0.782	-0.7 (9.4)	-3.9 (10.6)	0.257
	Intra-group p-value^2^	0.164	0.253	0.452		0.626	0.112	
	MCID, n (%)	13 (21.3%)	9 (18.8%)	4 (30.8%)	0.447	10 (24.4%)	3 (15.0%)	0.516
PCS-12 score	Preoperative, mean (SD)	42.0 (11.2)	41.5 (10.8)	43.6 (13.0)	0.418	42.9 (12.3)	39.9 (8.70)	0.205
	Postoperative, mean (SD)	45.5 (11.9)	46.8 (10.9)	40.5 (14.6)	0.169	45.8 (13.0)	44.8 (9.80)	0.275
	Delta^1^,mean (SD)	3.52 (10.3)	5.31 (9.83)	-3.1 (9.8)	0.013*	2.84 (10.3)	4.91 (10.6)	0.475
	Intra-group p-value^2^	0.010*	<0.001*	0.277		0.084	0.052	
	MCID, n (%)	29 (47.5%)	26 (54.2%)	3 (23.1%)	0.063	18 (43.9%)	11 (55.0%)	0.588
	Postoperative complications, n (%)	18 (29.5%)	14 (29.2%)	4 (30.8%)	1.000	13 (31.7%)	5 (25.0%)	0.810
	Second surgery, n (%)	4 (6.6%)	2 (4.17%)	2 (15.4%)	0.196	3 (7.3%)	0 (0.00%)	0.544
	Time to wound healing, days, mean (SD)	17.4 (15.1)	15.4 (6.51)	24.6 (29.6)	0.776	17.0 (13.1)	18.4 (19.1)	0.328

**Table 3:** Multivariate regression with delta PCS-12 as the dependent variable and aseptic versus septic bursitis as independent variables * and bold text indicate statistical significance (p<0.05) PCS: Physical Component Scale

Variable	Estimate	P-value	Lower 95	Upper 95
Aseptic	Reference			
Septic	-8.40	0.008*	-14.42	-2.38

For all patients undergoing olecranon bursa excision, there was a significant improvement in PCS-12 scores following surgery (preoperative: 42.0, postoperative: 45.5, p=0.010). On subgroup analysis, the aseptic group showed improvement in PCS-12 scores following surgery (preoperative: 41.5, postoperative: 46.8, p<0.001), while the septic group had statistically similar preoperative and postoperative scores (preoperative: 43.6, postoperative: 40.5, p=0.169). In the seven patients with rheumatoid arthritis, the average delta PCS-12 score was 9.57. More patients in the aseptic group met the MCID for PCS-12 (54.2% vs. 23.1%) but this difference did not achieve statistical significance (p=0.063). Overall, the patients did not experience a significant change in MCS-12 following surgery (-1.8; p=0.164). When comparing groups, there were no significant differences in preoperative, postoperative, or delta MCS-12 values. A similar number of patients met the MCID for MCS-12 (aseptic: 18.8%; septic: 30.8%; p=0.447) (Table 2). On multivariate regression analysis, a diagnosis of septic bursitis was independently associated with a worse outcome based on delta PCS-12 (estimate: -8.40, CI: -14.4 to -2.4, p=0.008) (Table 3).

Additional subgroup analysis was performed based on whether or not a concomitant triceps repair was performed. The patients with additional triceps repair patients were younger (mean age: 54.0 vs. 59.4 years, p=0.030) but had similar medical comorbidities and prior treatment (Table 1). No significant differences were found between these groups for delta PCS-12 (2.8 vs. 4.9, p=0.475) or delta MCS-12 (-0.7 vs. -3.9, p=0.257) scores. Neither group was found to have significant improvement after surgery based on PCS-12 (Table 2).

Patients had a statistically similar ROM before and after surgery (83.0% vs. 91.8%, p=0.228) when considering the whole cohort as well as the aseptic (78.4% vs. 89.5%, p=0.222) and septic (100% vs. 100%, p=1.000) subgroups. Patients receiving a triceps repair were less likely to have a full preoperative ROM (64.3% vs. 90.9%, p=0.040), but this group showed a significant improvement in the rate of patients with full ROM after surgery (64.3% vs. 100%, p=0.007).

Eighteen (29.5%) of the 61 patients experienced postoperative complications, including 10 patients with postoperative fluid collections, four patients with paresthesia in an ulnar nerve distribution, three patients with wound dehiscence, and one patient with impaired ROM (reduction of 30 degrees in supination and 5 degrees in extension). Of the 18 patients with complications, only one patient had a history of rheumatoid arthritis. Additionally, only two (11.1%) of the patients underwent partial bursectomy while all other patients underwent complete bursal excision. Four (6.5%) patients experienced a major complication including a return to the operating room for a second surgical procedure. One patient, in the aseptic group, reported recurrence of their olecranon bursitis symptoms and had a revision bursectomy 168 days after their index bursectomy. Three other patients, one in the aseptic group and two in the septic group, also required additional surgery. The patient in the septic group underwent an irrigation and debridement procedure 47 days after their index bursectomy for an ulcer with non-purulent drainage. The patient in the aseptic group underwent irrigation and debridement for recurrent accumulation of a seroma 70 days after their index bursectomy. The fourth patient, in the septic group, required plastic surgery to perform a skin graft for wound closure. The mean time to wound healing was 17.4 ±15.1 days, with no significant differences between subgroups. The patients with wound dehiscence achieved wound healing on days 26, 90, and 92.

## Discussion

Olecranon bursitis is a common condition that often leads to pain and disability in the affected elbow. Partial or complete open bursa excision as well as endoscopic bursa cleaning and resection are the established operative options for patients who do not respond to conservative therapy. Our cohort consisted of 61 patients with preoperative and one-year postoperative PCS-12 and MCS-12 documented, and we found a significant improvement in PCS-12 scores following surgery in the overall cohort, but this improvement was primarily found in the aseptic group.

Our study reported MCS-12 and PCS-12 as markers of patient-reported improvement following open olecranon bursectomy. We found that, on average, patients receiving operative treatment for their bursitis tend to perceive a physical health quality of life benefit from the surgery. This is especially true for patients diagnosed with aseptic bursitis as this cohort experienced an average improvement in their postoperative PCS-12 score of 5.3, while the septic group experienced an average decrease in their postoperative PCS-12 score of -3.1, which was reproduced on multivariate analysis and showed an average decrease in postoperative PCS-12 of -8.4. Additionally, while the findings regarding overall improvement in full ROM or postoperative extension lag were not statistically significant, this represents the first study to evaluate patient ROM before and after olecranon bursectomy. Other authors have reported improvement in patient-reported outcomes after endoscopic olecranon bursectomy. They observed that septic bursitis patients experienced greater improvement than aseptic patients when based on the visual analog scale for pain and the quick disabilities of the arm, shoulder, and hand scores [7]. These differences may be attributed to the different surgical techniques utilized between the studies as the soft tissue around the elbow is more tenuous posteriorly than anteriorly, especially when performing operations for inflammatory or infectious processes [10,11]. As a result, the endoscopic approach for septic olecranon bursitis may be the favorable technique for operative treatment due to its minimally invasive nature in an area of high soft tissue tension, although a direct comparison to an open technique has yet to be performed.

More patients in the aseptic group had improvement in PCS-12 that met the MCID compared to those in the septic group (54.2% vs. 23.1%). This finding did not achieve statistical significance (p=0.063). Given the lack of significance despite the large difference in those meeting the MCID, we assume that we were likely underpowered to detect a statistically significant difference between these groups. Our data do not show a difference between those meeting the MCID for improvement in terms of MCS-12 (p=0.447). Historically, surgical treatment for septic olecranon bursitis is thought to be indicated for refractory cases when no improvement is seen after one week of antibiotic therapy or when aspiration results in incomplete drainage [12]. However, new studies support the non-surgical management of septic bursitis and report similar outcomes between surgical and non-surgical management [5,13]. Furthermore, new research also suggests that treatment by antibiotics alone without bursal aspiration is sufficient to treat septic bursitis [14]. Although this treatment strategy may be optimal for some patients, this finding was based on a small cohort (n=19) and may not have been large enough to capture patients with septic bursitis that is refractory to conservative treatment. For those patients in whom bursectomy is indicated, our data suggest that patients with septic bursitis typically experience equivalent to worse outcomes based on PCS-12 (43.6 to 40.5, p=0.277) and MCS-12 (52.6 to 50.1, p=0.452) from the preoperative to the postoperative period. Although surgery can lead to a high rate of short-term complications, patients tend to achieve a statistically similar quality of life as their preoperative function by one year.

One potential confounder to our data is the presence of concomitant triceps repair leading to more improvement in terms of PCS-12 after surgery. The number of patients with olecranon bursitis and triceps rupture is surprising as this is a concomitant rupture and a rare complication and its incidence has not previously been reported in the literature [15-17]. Prior research has only described that individual cases of concomitant triceps rupture are associated with olecranon bursitis. However, as previous researchers have described chronic olecranon bursitis as a possible risk factor for the development of a distal triceps rupture, we felt that it was important to include these patients [18]. We postulate that triceps rupture may occur only in aseptic bursitis cases due to the chronic nature of aseptic bursitis or they may receive earlier treatment and therefore show no diagnostic findings of sepsis. However, on bivariate analysis, our data did not show that a concomitant triceps repair led to more improvement in terms of PCS-12 (p=0.475). The group with a triceps repair demonstrated more improvement in the proportion of patients with a full ROM after surgery. This finding may be attributed to the additional pain and swelling associated with the triceps tear limiting the patient’s ROM preoperatively [18]. Thus, when the pathology is resolved, their postoperative ROM evaluation is normal.

Our study showed a high postoperative complication rate of 29.5%, which was similar between groups (p=1.000). Other authors have reported complication rates ranging from 12.0% to 19.8% for surgical treatment of olecranon bursitis [19,20]. A systematic review on the topic suggested the complication rate to be 25.2% in surgically treated patients [12]. Additionally, previous research has demonstrated wound healing to be the most common complication experienced by patients undergoing surgery for olecranon bursitis. In our study, only three (16.7% of the individuals with a postoperative complication) patients experienced a wound healing complication. However, in a study by Degreef and De Smet, wound healing problems accounted for 27% of complications in patients undergoing surgery for olecranon bursitis [21]. It is unclear why our rate differs from these studies, but it may be due to differences in methodology for identifying postoperative complications, differences in postoperative documentation between institutions, or relative sizes of the study populations. Germawi et al. further elaborated on the complications by categories including hematomas (2%), non-healing wounds (8%), fistulas (1%), osteomyelitis (4%), and triceps tendon problems (13%). Our study found hematomas or seromas in 16.4% of our patients, wound complications in 4.9%, ulnar paresthesia in 6.6%, and impaired ROM in 1.6%. Despite a high complication rate, very few of the patients in our study required further operative intervention (6.6%). The study by Germawi et al. reported a revision surgery rate of 11.5% [19]. Others have reported revision rates of 4.5% for olecranon or prepatellar bursectomy after trauma [20], 10% after a single-stage bursectomy, and 16% after a two-stage bursectomy [22]. Only one of the patients (1.7%) in our study had bursitis refractory to bursectomy and required revision bursectomy. Despite a high complication rate, patients undergoing bursectomy were effectively treated for their bursitis symptoms. This finding is supported by Stewart et al., who demonstrated that patients without rheumatoid arthritis had a 94% rate of long-lasting symptomatic relief following olecranon bursectomy [19]. For septic bursitis, recurrence rates have been reported to be 15.6% in patients treated by bursectomy and antibiotic therapy at a median of 25.5 days [23]. We reviewed the medical records of our patients for one year after surgery and found that one additional patient (7.7%) in the septic group required additional irrigation and debridement 47 days after their initial procedure.

This study also documented patients with septic bursitis which is typically caused by direct trauma to the posterior elbow [2]. Skin flora then seeds the olecranon bursa through a transcutaneous process, which progresses as a result of poor vascularity of the olecranon bursa [23]. The diagnosis of septic bursitis in our cohort was predominately based on clinical examination and supplemented by culture data. A previous review has suggested that physical exam findings of tenderness, erythema, warmth, skin lesions, and fever are more likely associated with septic than aseptic bursitis [3]. Although these findings may also be indicative of a case of aseptic bursitis with a severe inflammatory response, previous survey results have suggested that 83% of providers differentiate between septic and aseptic bursitis by clinical presentation while 70% differentiated by bursal fluid culture [24]. In our study, 38.5% of the septic bursitis diagnoses were made with the inclusion of preoperative bursal fluid culture, and 53.8% of patients had positive culture data between preoperative and intraoperative studies. We classified the remainder of cases as septic bursitis based on the treating physician's clinical and intraoperative impression of the patient.

Limitations of our study include its retrospective design and limited sample size. Retrospective data were collected from electronic medical records, and this could be subject to bias. We relied on the diagnosis from physician records of aseptic versus septic olecranon bursitis, which may be subject to physician bias in diagnosis based on physical exams, radiographic studies, and laboratory workup. This methodology also may have resulted in the misclassification of some patients as septic when they were truly aseptic. However, due to the inherent limitations of medical records, we felt that this was the best available method to distinguish septic from aseptic bursitis and is supported by the findings of Baumbach et al. [25]. Furthermore, our sample size was limited due to this study requiring preoperative and postoperative patient-reported outcomes. However, this is still one of the largest studies to date investigating patient-reported outcomes following olecranon bursa excision. Additionally, a large number of patients in our study were not treated for isolated olecranon bursitis. We included patients with concurrent triceps tears, gout, and rheumatoid arthritis. Although all patients had significant olecranon bursitis, these concurrent conditions may confound the results. An MCID value has not been well established for the SF-12 in the treatment of specific elbow pathology. Instead, we utilized a well-established value for the shoulder. Lastly, it would have been ideal to compare patient-reported outcomes between patients undergoing surgery and those undergoing nonsurgical treatment, but since it is an orthopedic practice, many patients referred to our center had previously failed nonsurgical treatment.

## Conclusions

We aimed to address the scarcity of research on the surgical treatment of olecranon bursitis by analyzing patient-reported outcomes after olecranon bursectomy. We identified that patients with aseptic olecranon bursitis experienced improvement following olecranon bursectomy as measured by PCS-12. While there was an overall complication rate of 29.5% in our study, which is higher than the rate documented in prior literature, only 6.56% of our patients required additional surgery. Our findings suggest that patients with refractory olecranon bursitis treated with open olecranon bursa excision tend to gain a physical health benefit and therefore surgical treatment for this category of injury is a reasonable option.
